# Proinflammatory polarization of adipose tissue macrophages in cows with subclinical ketosis constitutes a critical driver of adipose tissue remodeling and inflammation

**DOI:** 10.1186/s40104-025-01252-3

**Published:** 2025-09-29

**Authors:** Bichen Zhao, Ming Li, Huijing Zhang, Renxu Chang, Jingyi Wang, Wanli Zhao, Yue Yang, Muhammad Usman, Juan J. Loor, Chuang Xu

**Affiliations:** 1 College of Veterinary Medicine, China Agricultural University, Beijing, 100193 China; 2 Mammalian NutriPhysio Genomics, Department of Animal Sciences and Division of Nutritional Sciences, University of Illinois, Urbana, 61801 USA

**Keywords:** Adipose tissue macrophages, Adipose tissue remodeling, Macrophage polarization, Subclinical ketosis

## Abstract

**Background:**

Sustained lipolysis exacerbates subclinical ketosis (SCK) in dairy cows and is associated with inflammation and adipose tissue macrophage (ATM) infiltration. While ATM involvement in adipose homeostasis and inflammation in early lactation is recognized, a comprehensive exploration of ATM polarization phenotypes in SCK cows is lacking. This study aimed to characterize ATM polarization and its link to lipolysis and inflammation in SCK cows.

**Results:**

Subcutaneous adipose tissue samples were obtained from dairy cows to analyze protein expression and gene profiles. Compared with healthy cows, SCK cows had higher serum BHBA and NEFA, smaller adipocytes, and increased expression of lipolytic enzymes (LIPE, ATGL), indicating enhanced lipolysis. Decreased levels of FASN, PPARγ, p-SMAD3, and TGFβ suggested impaired adipogenesis. Inflammatory markers (TNF-α, IFN-γ, TLR4, Caspase1) and NFκB signaling activity were elevated. ATM infiltration was supported by increased CD9, CD68, TREM2, and CXCL1 expression. Protein abundance of M1 polarization markers (iNOS, CD86 and CCL2) in ATMs were associated with greater levels of *NOS2*, *IL1B*, *CD86* and *CCL2* mRNA expression in SCK cows; fluorescence intensity of NOS2 and CD86 also was elevated, alongside a higher proportion of CD68^+^/CD86^+^ immunopositive cells within adipose tissue. ELISA further quantified increased concentrations of IL-1β and CCL2. Conversely, the abundance of ATM M2 polarization markers, including CD206, IL-10, KLF4, and Arg1, at both the protein and mRNA levels demonstrated a decline. Meanwhile, the proportion of CD68^+^/CD206^+^ immune response cells was relatively low in SCK cows.

**Conclusions:**

Overall, the present study indicated an augmented macrophage presence within adipose tissue during subclinical ketosis, with a predominance of pro-inflammatory macrophages (M1 ATM). This observation suggested a vicious cycle wherein macrophage infiltration and pro-inflammatory polarization coincide with enhanced lipolysis and an amplified inflammatory cascade.

**Supplementary Information:**

The online version contains supplementary material available at 10.1186/s40104-025-01252-3.

## Introduction

Ketosis is a metabolic disease with high economic impact to the dairy industry. Based on the measured blood concentration of β-hydroxybutyrate (BHBA), as well as changes in body condition score, appetite, milk production, and other clinical signs, ketosis in dairy cows can be categorized into clinical and subclinical (SCK) forms [1, 2]. The prevalence of clinical ketosis and SCK in large-scale, intensive farming operations ranges from 10% to 30% [3], with global annual losses estimated at approximately US$18 billion [4]. Ketosis is the most common periparturient disorder in high-producing dairy cows, particularly when cows experience severe negative energy balance (NEB) [5]. By releasing non-esterified fatty acids (NEFA) through lipolysis, adipose tissue plays a crucial role in regulating energy metabolism during NEB. Serum NEFA concentrations can serve as a reliable indicator of NEB, as they directly reflect the extent of adipose tissue breakdown [6].

During the first few weeks after calving, 30% to 50% of triacylglycerol (TAG) stored in adipocytes is mobilized to meet the energy demands of lactation, thereby sparing glucose for milk production [7, 8]. However, during severe NEB, excessive lipolysis leads to accumulation of NEFA, which may exceed the hepatic capacity for oxidation and metabolic clearance, thereby predisposing affected cows to ketosis and fatty liver [9, 10]. Plasma NEFA concentrations of 0.6 mmol/L during the first week postpartum are considered a predictive risk factor for ketosis [11]. These NEFA levels reflect a high rate of lipolysis and although recent studies have established a correlation between metabolic disorders in periparturient cows and lipid metabolism disorders associated with lipolysis, our understanding of specific therapeutic targets and cellular mechanisms regulating lipolysis remains limited.

Adipose tissue functions not only as an energy reservoir but also as an endocrine organ that regulates systemic metabolism and inflammatory processes. In both humans and rodents, intense lipolysis triggers an inflammatory response within adipose tissue [12]. This inflammatory response is partly mediated by adipose tissue macrophages (ATMs) that reside in or infiltrate the fat tissue [13]. In dairy cows, adipocyte volume decreases in the first few weeks postpartum due to increased lipolysis and reduced lipogenesis, which involves a remodeling process associated with inflammation [14, 15]. Adipose tissue inflammation is characterized by the accumulation of immune cells and increased inflammatory polarization, as well as the release of pro-inflammatory cytokines [16].

The ATMs can be broadly categorized into classical (M1) and alternative (M2) types, with the former being actively pro-inflammatory and the latter promoting inflammation resolution [17]. M1 ATMs secrete cytokines such as TNF-α and interleukins 1β and 6, which mediate local and systemic inflammation and inhibit the anti-lipolytic action of insulin in adipocytes [18, 19]. In contrast, the M2 phenotype is associated with healing and the resolution of inflammation, promoting the differentiation of new adipocytes (adipogenesis) and buffering excess free fatty acids (FFAs) through re-esterification into triglycerides. In addition, monocyte-derived macrophages recruited during metabolic disorders are also strongly associated with the extent of insulin resistance and metabolic disorders [20, 21]. Blocking recruited monocyte derived macrophages by targeting key monocyte chemokines has been reported to alleviate obesity-related inflammation and related metabolic disorders in mice, highlighting the role of macrophages in driving inflammation and affecting lipid homeostasis [22]. During their first lactation at day one postpartum, Akter et al. [23] observed an increase in ATM numbers in the omental and mesenteric fat depots of cows. The relationship between an inflammatory response of adipose tissue and ATM infiltration during intense lipolytic periods leading to SCK has not yet been assessed.

Excessive lipolytic conditions during NEB maintain a vicious cycle of chronic inflammation and imbalance of lipid metabolic states between immune cells and adipocytes in the adipose tissue [11, 24]. These contribute to increased susceptibility to infection and reduced immune response, which may be a key reason for the high prevalence of mastitis in ketosis cows [6]. Current interventions are insufficient to prevent the incidence of SCK in part because the early triggers that establish and maintain adipose tissue inflammation in early lactation remain elusive. Thus, elucidating the crosstalk between adipose tissue and ATM is urgently needed for the development of effective therapeutic strategies targeting excessive lipolysis and adipose inflammation. The present study aimed to evaluate ATM infiltration and their polarization phenotypes in SCK cows compared to healthy cows.

## Materials and methods

### Ethics statement

The experimental procedures adhered to the Guidelines for Laboratory Animal Care and Use established by the Chinese Center for Disease Control and Prevention. Ethical oversight was granted by the Ethics Committee for Animal Use and Care at China Agricultural University (Beijing, China; Approval No. AW21604202-2-1).

### Animals, diet*,* and experimental design

The experiment involved selecting Holstein dairy cows from a commercial 12,000 cow dairy farm located in Dingzhou City, Hebei Province, China. A total of 206 lactating Holstein cows were selected for testing from 21 d before the expected calving date to 14 d postpartum. Veterinarians evaluated body condition of cows using a 5-point scale [25]. Ketone concentrations in tail venous blood specimens were quantified utilizing a bovine-specific ketone analyzer (Yicheng Bioelectronics Technology Co. Ltd., Beijing, China). Cows in the dry period were monitored by assessing body condition score (BCS) and measuring blood BHBA at 21 d before the expected calving date. Cows with 3.0 ≤ BCS < 3.5 and BHBA < 1.0 mmol/L were selected for continued monitoring after parturition. Blood BHBA concentrations were further measured on d 5 and 10 postpartum. Dairy cows were classified based on blood BHBA concentrations measured on d 5 and 10 postpartum. Cows with BHBA levels below 1.0 mmol/L on both days were considered metabolically stable, whereas those with BHBA concentrations exceeding 1.4 mmol/L but below 2.8 mmol/L were categorized into the SCK group. All cows received routine medical checks to ensure they were free of other complications such as hypocalcaemia and mastitis. To ensure balanced group sizes, once 6 cows were enrolled in any group, additional cows meeting the same criteria were excluded from further inclusion.

Blood samples were collected before feeding by jugular venipuncture using anticoagulant-free blood collection tubes at 10–14 d after parturition (days in milk, DIM: median = 12 d, range = 10–14 d). Following a 2-h incubation at ambient temperature to facilitate coagulation, samples underwent centrifugation at 1,900 × *g* for 15 min at 4 °C to isolate serum. The concentrations of NEFA and BHBA in the serum were quantified using a Roche Cobas 6000 c501 automated biochemical analyzer (Guangzhou Zhanquan Biotechnology Co., Ltd.). Comprehensive details regarding the diet’s nutrient profile and the cows’ physiological indices are available in Table S1 (Additional file 1) and Table 1, respectively. All cows involved in this study were fed according to National Research Council recommendations during the dry and lactation periods (NRC, 2001) [26].

**Table 1:** The basic physiological parameters of the healthy and SCK cows

Parameter^1^	Average; SEM		*P*-value^2^
	Healthy cows (*n* = 6)	SCK cows (*n* = 6)	
Parity	2.83; 0.17	2.50; 0.22	0.664
BW, kg	618.83; 23.00	650.67; 19.34	0.030
BCS	2.79; 0.03	2.92; 0.07	0.072
DIM^3^	11.83; 0.60	11.33; 0.56	0.469
Milk yield, kg/d	37.87; 1.33	35.07; 1.49	0.048
DMI, kg/d	22.64; 0.80	20.85; 0.62	0.043

^1^*BW* Body weight, *DIM* Day in milk, *DMI* Dry matter intake

^2^*P*-value of the Student’s *t*-test

^3^Milk yield and DMI were calculated as the average values over the most recent 7 d for each cow

### Adipose tissue biopsy

Adipose tissue biopsy from the tail-head region was collected from each animal during the second week postpartum (10–14 DIM). Briefly, the tail-head and its surrounding hair were thoroughly cleaned with surgical soap. Local anesthesia with procaine hydrochloride (3%, 25 mL) was administered between the ischium and tailbone. Then, an incision (1.3–2.5 cm) parallel to the spine was made and the skin pulled using sterile hemostats to facilitate tissue collection. Adipose tissue samples weighing 1 to 2 g were obtained through blunt dissection using sterile forceps and surgical scissors. To achieve hemostasis and mitigate external hemorrhage, sterile gauze was applied for compressive pressure. The incision was secured with 6 to 8 surgical staples (Henry Schein). Harvested adipose specimens underwent rinsing with sterile isotonic saline solution (IN9000; Solarbio, Beijing, China) to eliminate residual contaminants. The biopsied tissues were either rapidly cryopreserved in liquid nitrogen or chemically stabilized using 4% paraformaldehyde (G1101; Servicebio, Wuhan, China) or embedded in an optimum cutting temperature (OCT) compound (G6059; Servicebio, Wuhan, China) for subsequent histological processing.

### Enzyme-linked immunosorbent assay (ELISA)

The cryopreserved adipose tissue, weighing approximately 200 mg, was immediately transferred to ice. Subsequently, the tissue underwent thorough mechanical disruption using an Ultrasonic Homogenizer (SCIENTZ-1ID, Ningbo Xinzhi Biotechnology Co. Ltd., Ningbo, China) operating at 400 Hz for 2 min within pre-cooled Hank’s Balanced Salt Solution. Upon completion of the homogenization process, the resultant mixture was subjected to centrifugal separation at 12,000 × *g* for 10 min at 4 °C, facilitating the precise retrieval of the supernatant.

The total protein content of the sample was quantified utilizing the bicinchoninic acid assay (P0011; Beyotime, Shanghai, China). The concentrations of LPS-binding protein (LBP), chemokine ligand 2 (CCL2), tumor necrosis factor-α (TNF-α), interleukin-1β (IL-1β), chemokine (C-X-C motif) ligand 1 (CXCL1), interferon-γ (IFNγ), and interleukin-10 (IL-10) in the adipose tissue homogenate were measured in accordance with the manufacturer’s guidelines using the respective commercially available ELISA kits (Bovine LBP ELISA kit: BPE92193; Bovine CCL2 ELISA kit: BPE92143; Bovine TNF-α ELISA kit: BPE92091; Bovine IL-1β ELISA kit: BPE92157; Bovine CXCL1 ELISA kit: BPE92218; Bovine IFNγ ELISA kit: BPE92141; Bovine IL-10 ELISA kit: BPE92159; Shanghai Langton Biotechnology Co. Ltd., Shanghai, China). Each group included six biological replicates and three technical replicates. Optical density measurements of sample absorbance were recorded at 450 nm utilizing a spectrophotometer (CMax Plus, Molecular Devices Instruments Co. Ltd., San Jose, CA, USA). Sensitivity of TNF-α, LBP, IFNγ, CXCL1, IL-1β, CCL2 and IL-10 as less than 2.5 ng/L, 0.272 μg/mL, 3 ng/L, 2.248 ng/L, 0.5 ng/L, 1.625 ng/L, 4 ng/L, respectively. Optical density measurements of sample absorbance were recorded at 450 nm utilizing a spectrophotometer (CMax Plus, Molecular Devices Instruments Co. Ltd., San Jose, CA, USA).

### Adipose tissue histology

For the formalin-fixed, paraffin-embedded specimens, adipose tissue was initially excised from 4% paraformaldehyde and meticulously trimmed before being transferred into dehydration vessels. A progressive dehydration regimen was subsequently implemented, utilizing a graded ethanol series followed by xylene immersion to facilitate exhaustive water removal. Upon achieving complete dehydration, the specimens underwent paraffin infiltration, embedding, and microtomy into 8-μm sections. These sections were maintained at ambient conditions until subsequent analyses. To ensure methodological uniformity, serial sections from the central region of each sample were systematically selected and subjected to specialized histochemical protocols to evaluate predefined morphological and molecular characteristics.

#### Hematoxylin and eosin staining

Tissue sections were processed through xylene and an ethanol gradient to achieve dewaxing and re-dehydration. Hematoxylin staining followed, enhancing nuclear visualization, while differentiation in 1% hydrochloric acid-alcohol and subsequent 0.6% ammonia-water treatment restored nuclear staining to blue. After rinsing, sections were dehydrated again and mounted in neutral gum. Under an optical microscope (BX51; Olympus Corporation, Tokyo, Japan), blue-stained nuclei and clear vacuoles representing lipid droplets were observed, and photomicrographs were captured with an attached camera (E-330; Corporation, Tokyo, Japan) for documentation.

#### Immunofluorescence staining

Formalin-fixed, paraffin-embedded tissue sections underwent deparaffinization and sequential dehydration following established protocols. Antigen retrieval was facilitated by immersing the sections in 10 mmol/L citric acid buffer (pH 6.0), subjecting them to thermal induction through microwave heating until boiling, and subsequently allowing them to equilibrate to ambient temperature. To mitigate nonspecific binding, a 3% bovine serum albumin solution was applied as a blocking reagent before incubating the sections overnight at 4 °C with primary antibodies. The primary antibodies employed in this study comprised TLR4 (1:1,000, bs-1021R, Bioss, Beijing, China), rabbit anti-NFκB polyclonal antibody (1:100; bs-0465R, Bioss), rabbit anti-CD9 polyclonal antibody (1:100; bs-2489R, Bioss, Beijing, China), rabbit anti-CD68 polyclonal antibody (1:100; bs-1432R, Bioss, Beijing, China), mouse anti-CD68 monoclonal antibody (1:100; NB600-985, NOVUS), rabbit anti-CD86 polyclonal antibody (1:100; bs-1035R, Bioss, Beijing, China), NOS2 (1:1,000; 8985-1-AP, Proteintech, St. Louis, MO, USA), rabbit anti-CD206 recombinant antibody (1:100; 81525-1-RR, Proteintech, Chicago, IL, USA), and rabbit anti-IL-10 polyclonal antibody (1:100; bs-6761R, Bioss, Beijing, China). Fluorophore-conjugated secondary antibodies, including FITC-labeled goat anti-mouse (1:400, Servicebio, Wuhan, China), CY3-conjugated goat anti-rabbit IgG (1:300, Servicebio, Wuhan, China), and CY3-conjugated goat anti-mouse IgG (1:300, Servicebio, Wuhan, China), were subsequently introduced to facilitate antigen detection. Finally, the sections were counterstained with a DAPI-containing mounting medium, and fluorescence microscopy (DMi8, Leica, Wetzlar, Germany) was employed for image acquisition.

### Western blotting

Adipose tissue samples (200 mg) were dissolved in 800 μL precooled (4 °C) lysis buffer (P0013B, Beyotime, Shanghai, China) containing phosphatase inhibitor (P1045-2, Beyotime, Shanghai, China), protease inhibitor (P1045-1, Beyotime, Shanghai, China), and phenylmethylsulfonyl fluoride (ST505, Beyotime, Shanghai, China) was homogenized by cryomilling using a tissue mixer mill (MM400; Retsch, Haan, Germany) at 30 Hz for 2 min. Adipose tissue homogenates underwent centrifugation at 4 °C and 15,000 × *g* for 10 min, after which the resultant supernatant was harvested as the total protein extract. Protein concentration was quantified utilizing the BCA assay kit (P0010, Beyotime, Shanghai, China). Subsequently, a 20 µg aliquot of protein was fractionated via Tris–glycine gel electrophoresis and electrotransferred onto polyvinylidene difluoride membranes (Millipore, Burlington, MA, USA). Membranes were then blocked with Tris-buffered saline containing Tween-20 (TBST) supplemented with 5% skim milk (BD-232100, BD Difco, Franklin Lakes, NJ, USA). For immunodetection, membranes were incubated overnight at 4 °C with primary antibodies targeting TLR4 (1:1,000; bs-1021R, Bioss), p-NFκB (1:150; bs-3485R, Bioss), NFκB (1:1,000; bs-0465R, Bioss), CD9 (1:1,000; bs-2489R, Bioss), CD68 (1:1,000; bs-1432R, Bioss), CD86 (1:1,000; bs-1035R, Bioss), KLF4 (1:1,000; bs-24533R, Bioss), p-IκB (1:1,000; AF2002, Affinity, Changzhou, China), IκB (1:1,000; AF5002, Affinity), IL-1β (1:1,000; AF4006, Affinity), PPARγ (1:800; WL01800, Wanlei, Shenyang, China), p-SMAD2/p-SMAD3 (1:800; WL02305, Wanlei, Shenyang, China), SMAD3 (1:500; WL02288, Wanlei), TGFβ1 (1:1,000; WL02998, Wanlei), TNFα (1:1,000; WL01581, Wanlei), NLRP3 (1:1,000; WL02635, Wanlei), Caspase 1 (1:1,000; WL03450, Wanlei), CCL2 (1:800; WL02966, Wanlei), IL-10 (1:1,000; WL03088, Wanlei), LIPE (1:1,000; 4107, CST, Danvers, MA, USA), p-LIPE (1:1,000; 4139, CST, Danvers, MA, USA), FASN (1:10,000; 10624-2-AP, Proteintech), ATGL (1:800; 55190-1-AP, Proteintech), NOS2 (1:1,000; 18985-1-AP, Proteintech, Chicago, IL, USA), CD206 (1:2,000; 81525-1-RR, Proteintech), and β-actin (1:3,000; AF7018, Affinity). Membranes were subsequently incubated for 45 min with either an anti-mouse secondary antibody (1:5,000; BA1050, Boster) or an anti-rabbit counterpart (1:5,000; BA1054, Boster, Pleasanton, CA, USA). Protein bands were visualized using Image Lab software (Bio-Rad Laboratories Inc., Hercules, CA, USA) in conjunction with an ultra-sensitive ECL chemiluminescent reagent (MAO186, Meilunbio, Wuhan, China). Densitometric analysis was performed with Image-Pro Plus 6.0 software.

### RT-qPCR analysis

Total RNA was isolated from adipose tissue utilizing the Trizol reagent (Takara Biotech) following the manufacturer’s protocol. Subsequently, 5–10 µg of total RNA was solubilized in 10–20 µL of RNase-free water. Quantification of RNA concentration was conducted using a NanoDrop 2000 spectrophotometer (Thermo Fisher Scientific, Waltham, MA, USA). In alignment with the Minimum Information for Publication of Quantitative Real-Time PCR guidelines [27], RNA integrity was evaluated based on the optical density ratio at 260/280 nm, with a benchmark range of 1.8 to 2.0 indicative of acceptable purity. The 260/280 nm ratio of all samples was 1.8 to 2.0. The data of RNA integrity measured by the Agilent 2100 bioanalyzer indicated that RNA integrity number of all samples was above 8.0. The All-in-One First-Strand cDNA Synthesis SuperMix for qPCR (AE341-02; TransGen, Beijing, China) was then used to synthesize complementary DNA from total RNA samples. A PerfectStart® Green qPCR SuperMix (AQ602-03; TransGen, Beijing, China) was employed to quantify mRNA levels within the sample using an Applied Biosystems 7500 Real-Time PCR System. The relative abundance of mRNA was calibrated against the geometric mean of Ct values for β-actin and GAPDH and subsequently determined through the 2^−ΔΔCt^ algorithm. Primer sequences are detailed in Table S2.

### Image analysis

Photomicrographs for each experimental condition were obtained from a minimum of five biological replicates—distinct animal-derived samples analyzed under identical experimental parameters—across normal, mild, and moderate groups. Additionally, three technical replicates, representing repeated measurements from the same biological sample, were conducted per biological replicate. Adipocyte diameter was quantified using ImageJ software integrated with the adipocyte tools plugin. For immunofluorescence analysis, five independent fields per technical replicate were examined. Fluorescence intensity of TLR4 (threshold range: 23–160), NFκB (40–160), CD9 (48–255), CD68 (55–255), CD86 (30–255), NOS2 (58–255), CD206 (62–255), and IL-10 (10–255) was quantified via ImageJ. Furthermore, the proportion of CD68^+^/CD86^+^-positive and CD68^+^/CD206^+^-positive cells—calculated as the percentage of CD68^+^/CD86^+^- or CD68^+^/CD206^+^-expressing cells relative to total DAPI-stained nuclei within each field of view—was subjected to statistical evaluation.

### Statistical analysis

The dataset underwent statistical evaluation utilizing GraphPad Prism (version 8.0.0, GraphPad Software). Student’s *t*-test was performed in SPSS 26.0 (IBM Corp.) software to statistically compare the two groups in terms of basic information, serum biochemical indicators and gene expression levels. The results expressed as means ± standard error of the mean (SEM). A *P* < 0.05 was deemed statistically significant, and a *P* < 0.01 was considered highly significant.

## Results

### Adipose histopathological analysis, and adipogenic-, differentiation- and lipolysis-related proteins in adipose tissue of cows with SCK

**Fig. 1:**
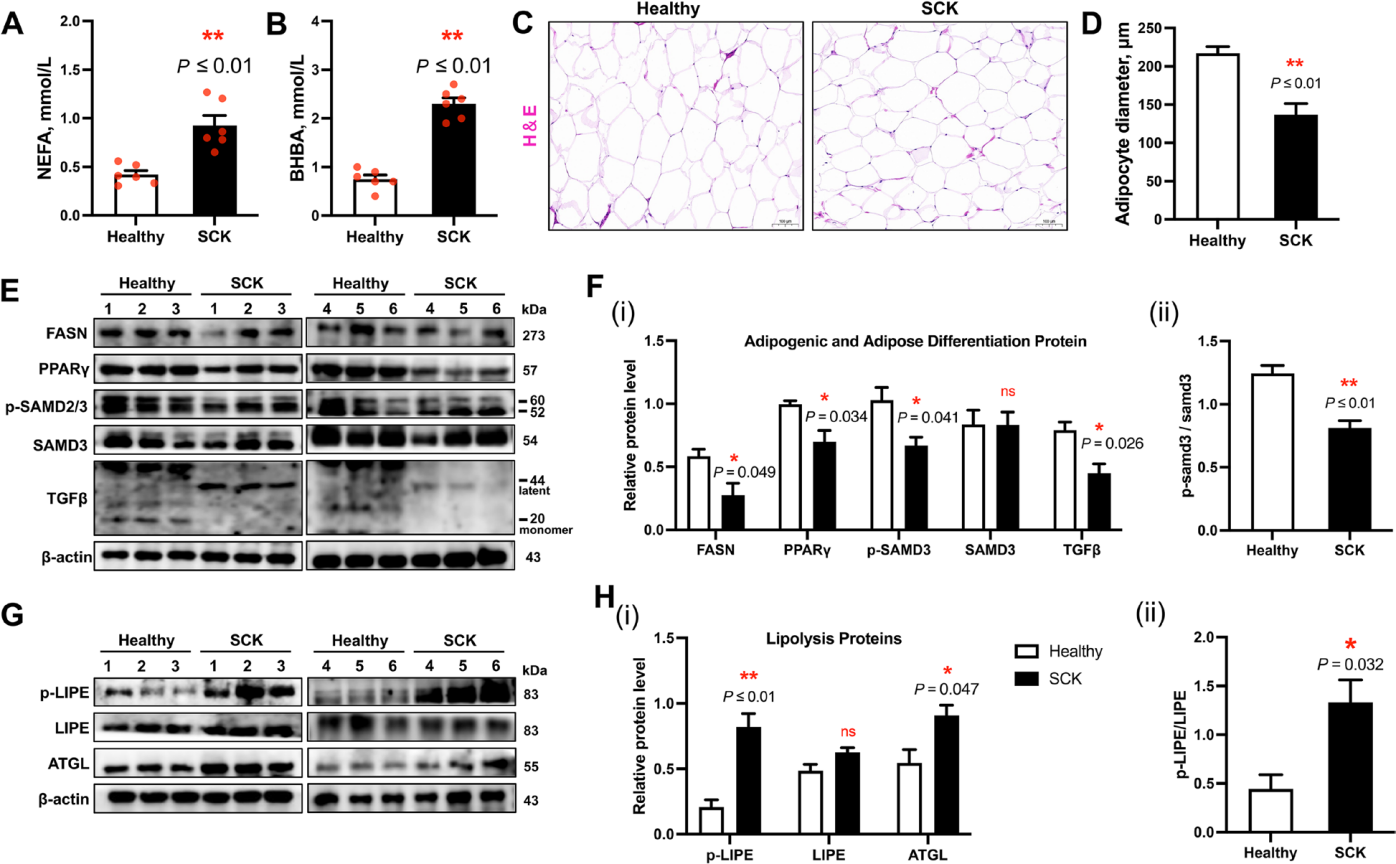
Blood parameters, adipose tissue histomorphology analysis and adipose tissue metabolism in healthy and SCK cows. **A** Serum concentrations of nonesterified fatty acid (NEFA). **B** Serum concentrations of β-hydroxybutyrate (BHBA). **C** Representative images of hematoxylin and eosin (H&E; scale bar 100 μm) staining of adipose tissue sections. **D** Quantitative analysis of adipose tissue diameter in healthy (*n* = 6) and SCK (*n* = 6) cows. **E** Representative Western blots of fatty acid synthase (FASN), peroxisome proliferative activated receptor gamma (PPARγ), phosphorylated mothers against decapentaplegic homolog 2/3 (p-SMAD2/3), SMAD3, transforming growth factor beta (TGFβ), and β-actin in the liver of healthy (*n* = 6) and SCK (*n* = 6) cows. **F** (i) Quantification of protein levels of FASN, PPARγ, p-SMAD2/3, SMAD3 and TGFβ. (ii) The ratios of p-SMAD3/SMAD3. **G** Representative Western blots of phosphorylated lipase E hormone sensitive type (p-LIPE), LIPE, adipose triglyceride lipase (ATGL), and β-actin in the liver of healthy (*n* = 6) and SCK (*n* = 6) cows. **H** (i) Quantification of protein levels of p-LIPE, LIPE and ATGL. (ii) The ratios of p-LIPE/LIPE. Data are presented as mean ± SEM, ^*^P < 0.05, ^**^P < 0.01; statistical differences were assessed by *t*-test

In comparison to their healthy counterparts, subclinical ketosis (SCK) cows exhibited elevated serum levels of NEFA (0.93 vs. 0.42 mmol/L) and BHBA (2.3 vs. 0.75 mmol/L), with statistical significance (*P* ≤ 0.01) (Fig. 1A and B). Histological examination via hematoxylin and eosin staining revealed a reduction in adipocyte diameter, indicative of intensified lipolytic activity in SCK-affected cattle (*P* ≤ 0.01; Fig. 1C and D). Moreover, immunoblot analysis of adipose tissue demonstrated reduced protein abundance of fatty acid synthase (FASN) (*P* = 0.049), peroxisome proliferator-activated receptor gamma (PPARγ) (*P* = 0.034), phosphorylated SMAD3 (p-SMAD3) (*P* = 0.041), and transforming growth factor-beta (TGFβ) (*P* = 0.026), alongside a diminished p-SMAD3/SMAD3 ratio in SCK cows relative to healthy controls (*P* ≤ 0.01) (Fig. 1E and F). In addition, phosphorylation of LIPE (which led to greater p-LIPE/LIPE) (*P* = 0.032) and protein abundance of ATGL (*P* = 0.047) were greater in the adipose tissue of SCK cows compared to controls (Fig. 1G and H).

### Activation of inflammatory signaling pathways in adipose tissue of cows with SCK

**Fig. 2:**
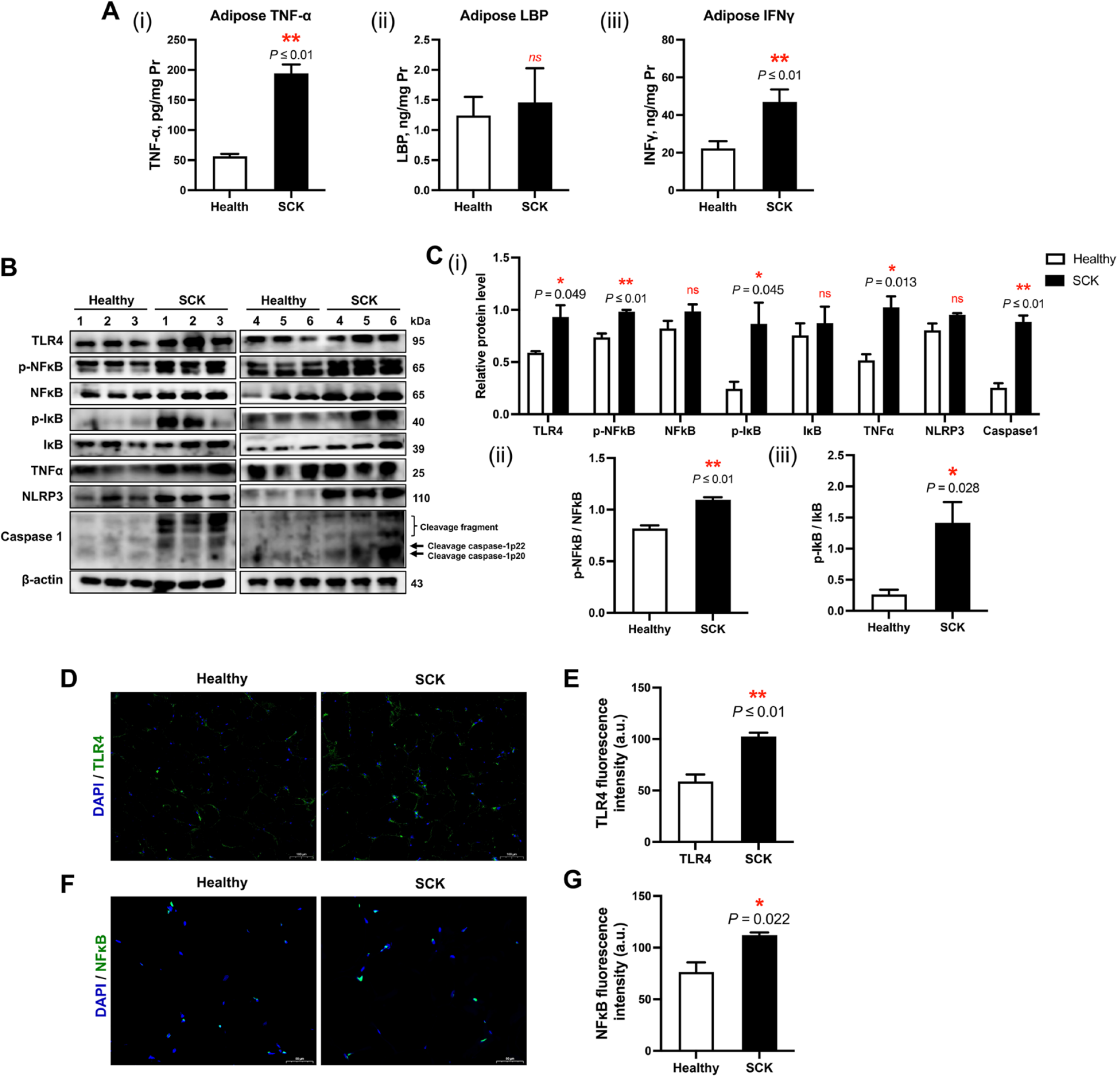
Adipose tissue inflammatory response in healthy cows and SCK cows. **A** The concentrations of (i) tumor necrosis factor (TNF)-α, (ii) lipopolysaccharide binding protein (LBP), (iii) interferon-gamma (IFN-γ) in adipose tissue homogenate of healthy (*n* = 6) and SCK group (*n* = 6). **B** Representative Western blots of toll-like receptor 4 (TLR4), phosphorylated nuclear factor kappa B subunit (p-NFκB), NFκB, phosphorylated inhibitor of nuclear factor kappa B kinase subunit beta (p-Iκb), Iκb, TNF-α, NLR Family Pyrin Domain Containing 3 (NLRP3), Caspase1 and β-actin in the adipose tissue of healthy (*n* = 6) and SCK (*n* = 6) cows. **C** (i) Quantification of protein levels of TLR4, p-NFκB, NFκB, p-Iκb, Iκb, TNF-α, NLRP3 and Caspase1. The ratios of (ii) p-NFκB/NFκB, (iii) p-Iκb/Iκb. **D** Representative images (scale bar = 100 μm) of immunofluorescence for TLR4 (green) and DAPI (blue). **E** Quantification of TLR4 fluorescence intensity (a.u.) in the adipose tissue of healthy (*n* = 6) and SCK (*n* = 6) cows. **F **Representative images (scale bar = 50 μm) of immunofluorescence for NFκB (green) and DAPI (blue). **G** Quantification of NFκB fluorescence intensity (a.u.) in the adipose tissue of healthy (*n* = 6) and SCK (*n* = 6) cows. Data are presented as mean ± SEM, ^*^P < 0.05, ^**^P < 0.01; statistical differences were assessed by *t*-test

The levels of TNF-α and IFN-γ in the adipose tissue of SCK cows were 194.16 ± 14.91 pg/mg protein (*P* ≤ 0.01) and 46.96 ± 6.68 ng/mg protein (*P* ≤ 0.01), respectively, which were significantly higher than those in healthy cows (TNF-α: 56.23 ± 4.11 pg/mg protein; IFN-γ: 22.29 ± 3.83 ng/mg protein) (Fig. 2A). Compared with healthy cows, SCK cows exhibited elevated protein abundance levels of TLR4 (*P* = 0.049), TNF-α (*P* = 0.013), and Caspase1 (*P* ≤ 0.01), alongside increased phosphorylation ratios of NFκB (p-NFκB/NFκB) and inhibitor of IκB (p-IκB/IκB) (Fig. 2B and C). Aligning with these protein quantification findings, immunofluorescence signal intensities for TLR4 (*P* ≤ 0.01) and NFκB (*P* = 0.022) were also greater in SCK cows (Fig. 2D–G).

### Infiltration of ATM in adipose tissue of cows with SCK

**Fig. 3:**
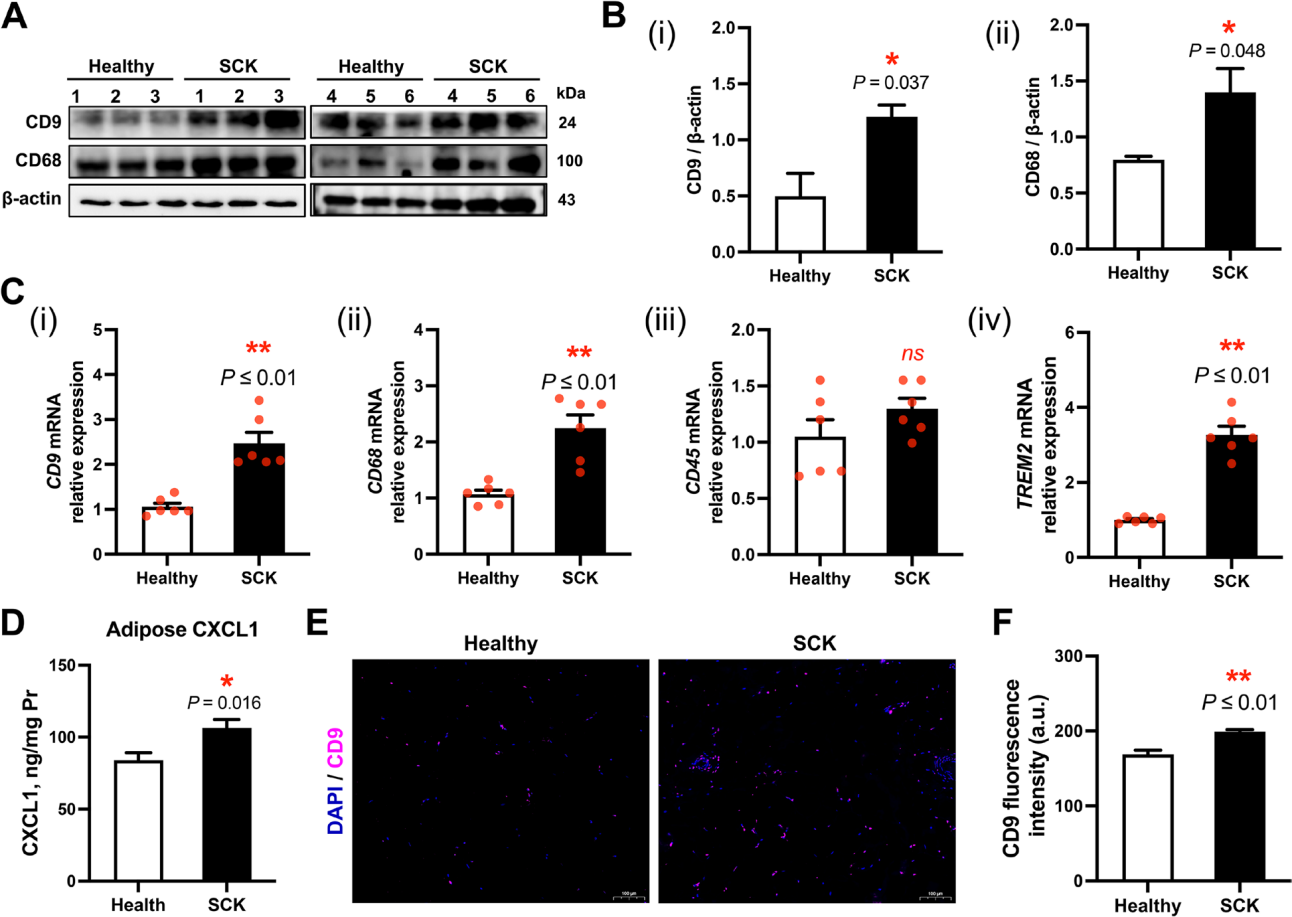
Adipose tissue macrophage counts and associated genetic changes in healthy cows and SCK cows. **A** Representative Western blots of cluster of differentiation 9 (CD9), CD68 and β-actin in the adipose tissue of healthy (*n* = 6) and SCK (*n* = 6) cows. **B** Quantification of protein levels of CD9 and CD68. **C** Relative mRNA expression levels of (i) *CD9*, (ii) *CD68*, (iii) *CD45* and (iv) triggering receptor expressed on myeloid cells 2 (*TREM2*) in adipose tissue of healthy and SCK groups (*n* = 6). **D** The concentrations of C-X-C motif chemokine ligand 1 (CXCL1) in adipose tissue homogenate of healthy and SCK groups (*n* = 6). **E** Representative images (scale bar = 100 μm) of immunofluorescence for CD9 (pink) and DAPI (blue). **F** Quantification of CD9 fluorescence intensity (a.u.) in the adipose tissue of healthy (*n* = 6) and SCK (*n* = 6) cows. Data are presented as mean ± SEM, ^*^P < 0.05, ^**^P < 0.01; statistical differences were assessed by *t*-test

The protein abundance levels (*P* ≤ 0.05) and transcriptional expression (*P* ≤ 0.01) of the ATM markers CD9 and CD68 were significantly elevated in SCK cows relative to healthy cows (Fig. 3A–C). Additionally, *TREM2* mRNA level was significantly elevated in adipose tissue of SCK cows (*P* = 0.022; Fig. 3C). The concentration of macrophage chemokine CXCL1 in adipose tissue was 106.41 ± 5.79 ng/mg Pr, which was significantly greater compared with healthy cows (84.00 ± 5.20 ng/mg Pr) (*P* = 0.016; Fig. 3D). The immunofluorescence staining analysis indicated an elevated fluorescence intensity of CD9 in the adipose tissue of SCK dairy cows (*P* = 0.022; Fig. 3C), consistent with the observed protein expression and mRNA relative abundance (Fig. 3E and F).

### ATM in adipose tissue of cows with SCK are predominantly M1-polarised

**Fig. 4:**
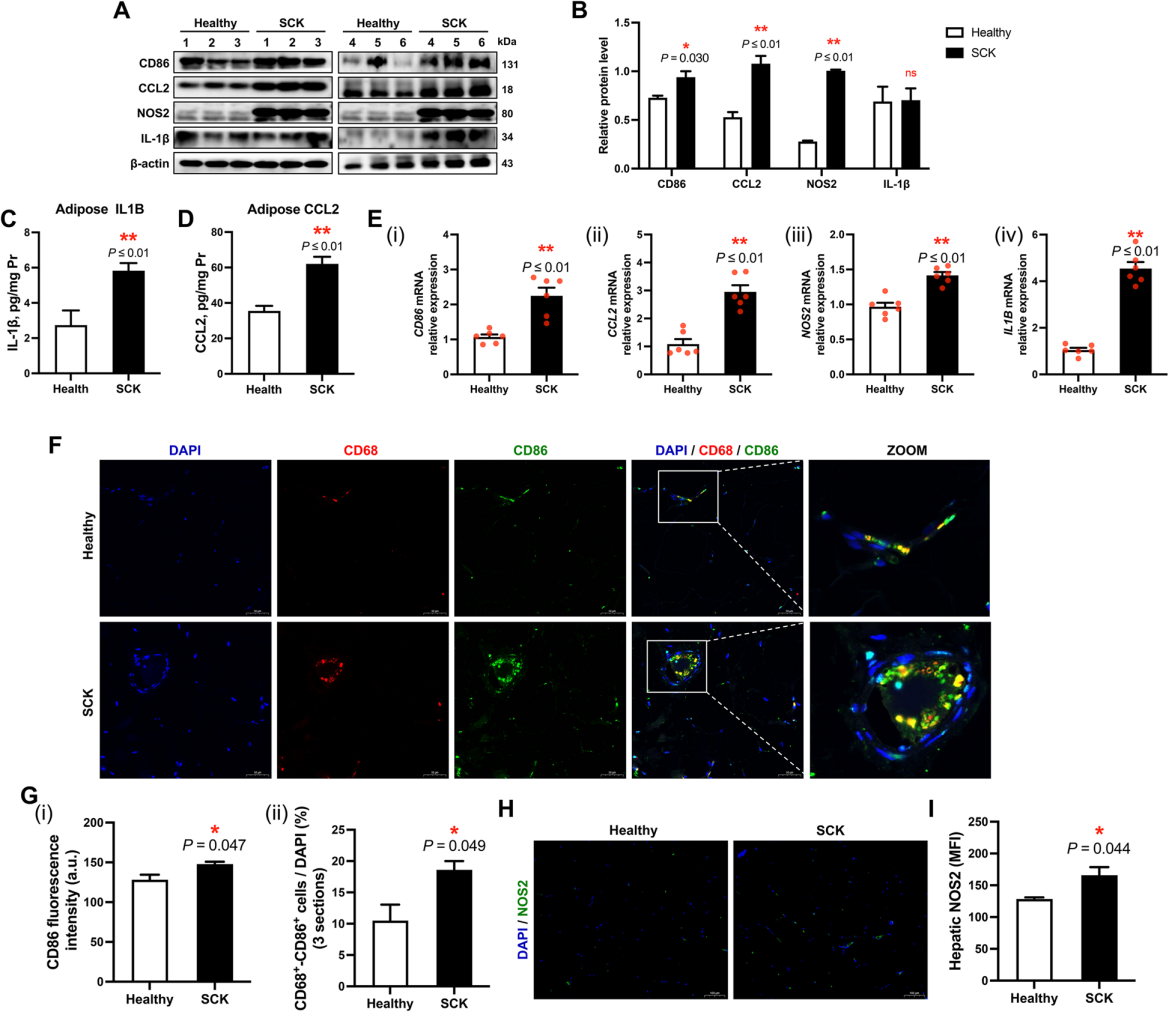
Proinflammatory polarization (M1-polarization) of adipose tissue macrophages in healthy cows and SCK cows. **A** Representative Western blots of CD86, C–C motif chemokine ligand 2 (CCL2), inducible nitric oxide sythase (iNOS), interleukin 1 beta (IL-1β) and β-actin in the adipose tissue of healthy (*n* = 6) and SCK (*n* = 6) cows. **B** Quantification of protein levels of CD86, CCL2, NOS2 and IL-1β. The concentrations of IL-1β (**C**) and CCL2 (**D**) in adipose tissue homogenate of healthy (*n* = 6) and SCK group (*n* = 6). **E** Relative mRNA expression levels of (i) *CD86*, (ii) *CCL2*, (iii) *NOS2* and (iv) *IL1B* in adipose tissue of healthy (*n* = 6) and SCK group (*n* = 6). **F** Representative images (scale bar = 50 μm) of immunofluorescence for CD68 (red), CD86 (green) and DAPI (blue) in bovine adipose tissue. **G** (i) Quantification of CD86 fluorescence intensity (a.u.) in the adipose tissue of healthy (*n* = 6) and SCK (*n* = 6) cows. (ii) Quantification of D68^+^/CD86^+^ positive cells (as a proportion of total DAPI) in the adipose tissue of healthy cows (*n* = 6) and SCK cows (*n* = 6). **H** Representative images (scale bar = 100 μm) of immunofluorescence for NOS2 (green) and DAPI (blue). **I** Quantification of NOS2 fluorescence intensity (a.u.) in the adipose tissue of healthy (*n* = 6) and SCK (*n* = 6) cows. Data are presented as mean ± SEM, ^*^P < 0.05, ^**^P < 0.01; statistical differences were assessed by *t*-test

The protein abundance of M1 polarization indicators in ATMs, namely CD86 (*P* = 0.030), CCL2 (*P* ≤ 0.01) and NOS2 (*P* ≤ 0.01), was elevated in the adipose tissue of SCK cows relative to healthy cows (Fig. 4A and B). Compared with healthy cows (IL-1β: 2.74 ± 0.83 pg/mg Pr; CCL2: 35.50 ± 2.86 pg/mg Pr), the concentrations of IL-1β and CCL2 in adipose tissue of SCK cows were higher, 5.83 ± 0.44 pg/mg Pr (*P* ≤ 0.01) and 62.09 ± 3.95 pg/mg Pr (*P* ≤ 0.01), respectively (Fig. 4C and D). In addition, greater mRNA expression levels of *CD86* (*P* ≤ 0.01), *CCL2* (*P* ≤ 0.01), *NOS2* (*P* ≤ 0.01) and *IL1B* (*P* ≤ 0.01) also were observed in adipose tissue of SCK cows (Fig. 4E). Immunofluorescence analysis corroborated the findings from both protein and mRNA assessments, demonstrating an elevated fluorescence signal for CD86 (*P* = 0.047) and NOS2 (*P* = 0.044) within the adipose tissue of SCK cows relative to healthy cows (Fig. 4F–I). Notably, a pronounced M1 polarization phenotype of ATM was identified in SCK cows, as evidenced by an increased proportion of CD68^+^/CD86^+^ immunopositive cells (*P* = 0.049, Fig. 4F and G).

**Fig. 5:**
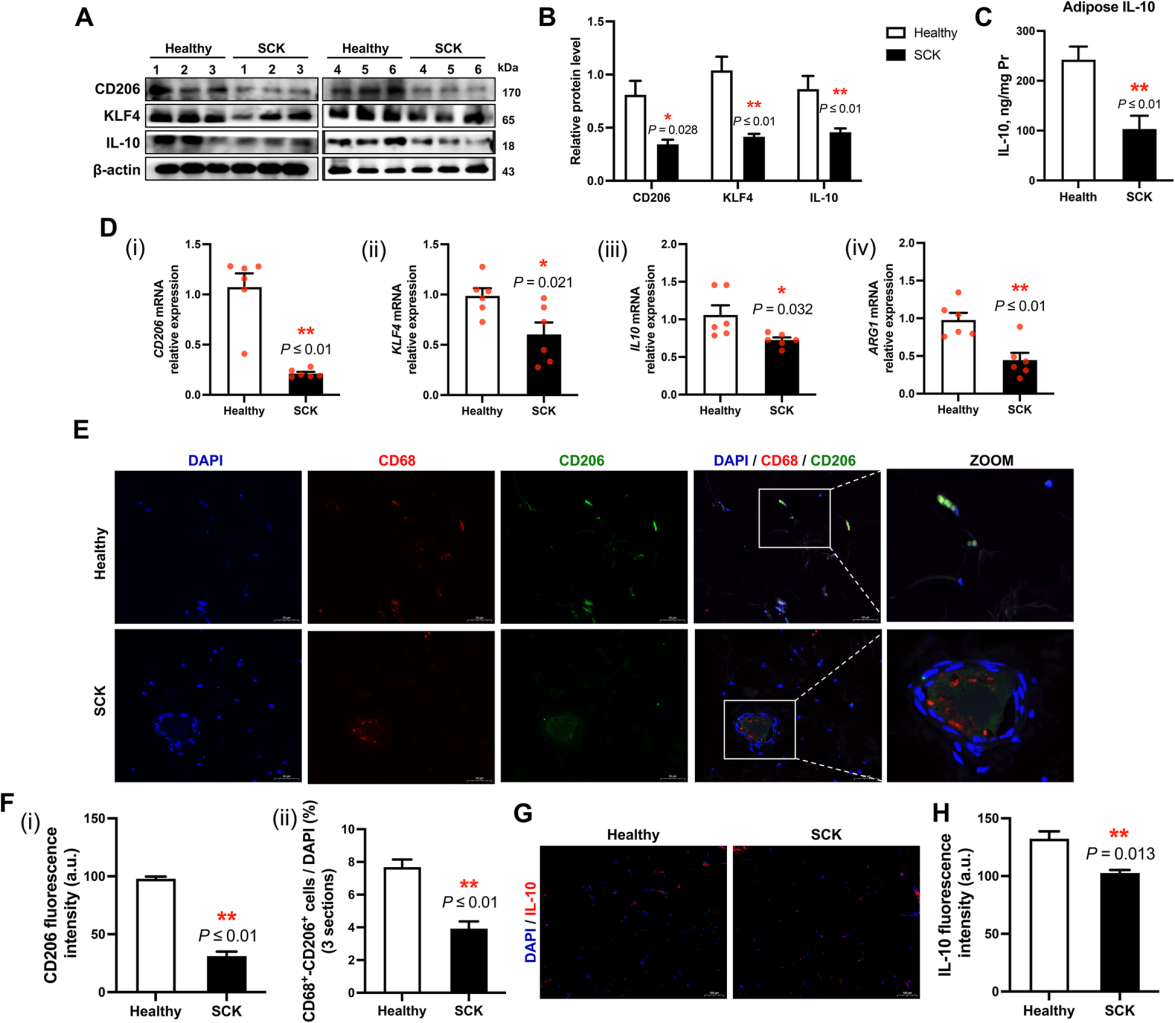
Anti-inflammatory polarization (M2-polarization) of adipose tissue macrophages in healthy cows and SCK cows. **A** Representative Western blots of CD206, krueppel-like factor 4 (KLF4), IL-10 and β-actin in the adipose tissue of healthy (*n* = 6) and SCK (*n* = 6) cows. **B** Quantification of protein levels of CD206, KLF4 and IL-10. **C** The concentrations of IL-10 in adipose tissue homogenate of healthy and SCK groups (*n* = 6). **D** Relative mRNA expression levels of (i) *CD206*, (ii) *KLF4*, (iii) *IL10* and (iv) Arginase 1 (*Arg1*) in adipose tissue of healthy and SCK groups (*n* = 6). **E** Representative images (scale bar = 50 μm) of immunofluorescence for CD68 (red), CD206 (green) and DAPI (blue) in bovine adipose tissue. **F** (i) Quantification of CD206 fluorescence intensity (a.u.) in the adipose tissue of healthy (*n* = 6) and SCK (*n* = 6) cows. (ii) Quantification of D68^+^/CD206^+^ positive cells (as a proportion of total DAPI) in the adipose tissue of healthy cows (*n* = 6) and SCK cows (*n* = 6). **G** Representative images (scale bar = 100 μm) of immunofluorescence for IL-10 (red) and DAPI (blue). **H** Quantification of IL-10 fluorescence intensity (a.u.) in the adipose tissue of healthy (*n* = 6) and SCK (*n* = 6) cows. Data are presented as mean ± SEM, ^*^P < 0.05, ^**^P < 0.01; statistical differences were assessed by *t*-test

The expression levels of ATM M2 polarization markers, specifically CD206 (*P* = 0.028), KLF4 (*P* ≤ 0.01), and IL-10 (*P* ≤ 0.01), exhibited a significant reduction in adipose tissue of SCK cows (Fig. 5A and B). The IL-10 concentration within adipose tissue of the SCK group was quantified at 103.28 ± 26.54 ng/mg Pr, markedly lower than the 242.24 ± 26.32 ng/mg Pr observed in healthy cows (*P* ≤ 0.01, Fig. 5C). Additionally, transcriptional activity of *CD206* (*P* ≤ 0.01), *KLF4* (*P* = 0.021), *IL10* (*P* = 0.032), and *ARG1* (*P* ≤ 0.01) demonstrated a similar downregulation in SCK cows (Fig. 5D). Immunofluorescence assessments corroborated these molecular alterations, as evidenced by diminished fluorescence intensity of CD206 (*P* ≤ 0.01) and IL-10 (*P* = 0.013), alongside a decreased proportion of CD68^+^/CD206^+^ double-positive cells within adipose tissue of SCK cows (*P* ≤ 0.01, Fig. 4E–G).

## Discussion

The transition period in dairy cows is distinguished by alterations in dry matter intake (DMI) and energy balance, which impact the rates of adipose synthesis and mobilization [28]. Cows with ketosis are distinguished by a high rate of lipolysis; excessive fat breakdown increased susceptibility to disease and affects negatively lactation performance [29]. As recently documented in rodent and human models, lipid catabolism within adipose tissue initiates a restructuring process distinguished by the recruitment of ATM and the subsequent emergence of inflammatory processes [30, 31]. Given the crosstalk between lipolysis and inflammatory events in adipose tissue, our current study aimed to quantify lipolysis, synthesis, inflammatory responses, and ATM infiltration in subcutaneous adipose tissue of SCK cows. The aim was to generate evidence for the crosstalk among these factors in SCK cows that could help explain the prolonged and severe lipolysis observed during the course of SCK in dairy cows.

Macrophages constitute the predominant immune cell population within bovine adipose tissue and are recognized as principal mediators of sustained inflammatory responses linked to lipid mobilization [32, 33]. In dairy cows, ATM infiltration occurs as a response to intense lipolytic activity [34]. In rodent models where lipolysis is induced, macrophages infiltrate adipose tissue in response to the increased release of NEFA by adipocytes [35]. Previous research has documented the infiltration of macrophages within the omental and subcutaneous adipose tissues of periparturient dairy cows experiencing abdominal displacement [15]. In circumstances characterized by elevated free fatty acid (FFA) mobilization—such as metabolic disorders, feed restriction, and the postpartum period—there is a notable increase in the number of ATM and the expression of genes associated with ATM infiltration in comparison to control cows [28]. This finding aligns with our study, which observed extensive ATM recruitment in the subcutaneous adipose tissue of SCK cows, manifested by high expression of CD68 and CD9, with ATMs forming crown-like structures around adipocytes.

The dynamic nature of ATMs allows them to respond to changes in the specific adipose tissue environment and to exhibit varying activation states, such as M1 and M2 polarization [36]. As initially demonstrated in non-ruminants, lipolysis can modulate macrophage phenotypes, e.g., NEFA and other lipolytic by-products serve as potent activators for the pro-inflammatory polarization (M1 phenotype) of ATMs [37, 38]. Thus, the observed increase of M1 and decrease of M2 macrophages in subcutaneous adipose tissue of SCK cows can be partly attributed to the direct induction by elevated NEFA levels. In turn, by forming a microenvironment enriched in IL1β, TNF-α, and IFN-γ, the activation of pro-inflammatory ATMs induces metabolic inflammation and establishes a crosstalk with adipocytes, which as suggested earlier plays a role in regulating lipolysis [39].

Our findings confirm that cows with SCK are actively mobilizing lipid from adipose tissue, as evidenced by elevated levels of circulating NEFA and BHBA, alongside a reduction in adipocyte size. Furthermore, the increased expression of pivotal lipolytic enzymes, such as hormone-sensitive lipase (LIPE) and adipose triglyceride lipase (ATGL), further indicates an enhanced lipolytic process, in line with the observations reported by Xu et al. [40]. These results underscore that ATM in SCK cows are more polarized towards the M1 phenotype, concomitant with upregulated pro-inflammatory cytokine expression and exacerbated lipid catabolism. This observation implies a reciprocal interaction between macrophage polarization and adipocyte lipolysis.

During adipose tissue remodeling in adult animals, the resident M2 ATM play a pivotal role in supporting adipogenesis. By releasing osteopontin, the M2 ATM trigger signals for adipocyte proliferation and the formation of new adipogenic foci [41, 42]. Adipogenesis in dairy cows also adapts to the energy demands of different production stages, where the end of lactation and the dry period constitute stages during which adipose tissue stores energy as TAG in adipocytes (increased adipogenesis), and this energy is released during NEB (decreased adipogenesis) [6, 43]. The diminished expression of fatty acid synthase (FASN), peroxisome proliferator-activated receptor gamma (PPARγ), and transforming growth factor-beta/small mother against decapentaplegic (TGF-β/SMAD) signaling pathways in the adipose tissue of SCK cows indicated a potential attenuation of adipogenic processes, which may be partially attributable to the decreased prevalence of M2 ATM.

The ATMs serve as an immune metabolic regulator in adipose tissue, with the inflammatory response in AT of cows with subclinical ketosis (SCK) being explained from two perspectives: firstly, the high lipolytic rate of adipocytes can trigger local lipotoxicity; secondly, the effect of ATM on inflammatory polarization [6, 44]. The CCL2 and CXCL1 produced by ATM recruit a large number of macrophages, which may then tend to M1 polarize under FFA stimulation. Furthermore, the pro-inflammatory mediators produced by M1 ATMs are key effectors in the inflammatory signaling and impairment of adipocyte function, potentially inducing more severe local inflammation [45, 46]. Such events were demonstrated in the present study by the greater protein abundance of components in the TLR4-NFkB and the NLRP3-caspase 1 pathways along with elevated levels of pro-inflammatory factors in the adipose tissue of SCK dairy cows. Thus, these data emphasize the potential role of increased macrophage infiltration and the enhancement of the pro-inflammatory phenotype in promoting inflammation within adipose tissue.

Most studies investigating the inflammatory processes associated with lipolysis in SCK cows have concentrated on gene expression profiles [6, 47]. As mentioned earlier, the number of macrophages in subcutaneous AT of SCK cows is high [48, 49], but data on macrophage phenotypes are lacking. In the present study, the polarizing infiltration of M1 ATM in subcutaneous adipose tissue of cows with ketosis coincided with an enhanced local inflammatory response and strong adipose tissue remodeling. Thus, along with published data, the present study supports the hypothesis that infiltration of SCK dairy cow macrophages into AT enhances lipolysis forming a vicious cycle linking lipolysis, ATM infiltration, and inflammation.

As described in human and mouse studies focused on ways to alleviate local inflammation by drug-induced adipose tissue anti-inflammatory (M2) macrophage phenotype [44–46], the possibility of modulating ATM phenotype in SCK cows may provide therapeutic options given the strong polarization of ATM to M1 during hyperlipolysis [17].

## Conclusions

Infiltration of macrophages in adipose tissue and the formation of coronial structures around the cells with a higher proportion of pro-inflammatory (M1) macrophages are characteristics of the subclinically ketotic state. This phenomenon may act as a critical trigger for enhanced lipolysis and inflammatory responses, establishing a vicious cycle that links lipolysis, adipose tissue macrophage (ATM) infiltration and inflammation. The role of macrophage polarization in the prolonged and severe lipolytic processes observed in SCK dairy cows warrants further investigation, which will improve herd health and profitability.

## Supplementary Information

Additional file 1: Table S1 Ingredient and nutrient composition of the diets. Table S2 Primer sequences of the genes.

## Data Availability

The datasets used during the current study are available from the corresponding author on reasonable request.

## Abbreviations

ARG1: Arginase 1
ATGL: Adipose triglyceride lipase
ATM: Adipose tissue macrophage
BHBA: β-Hydroxybutyrate
BW: Body weight
CCL2: C-C motif chemokine ligand 2
CD9: Cluster of differentiation 9
CD68: Cluster of differentiation 68
CD86: Cluster of differentiation 86
CD206: Cluster of differentiation 206
CXCL1: Chemokine (C-X-C motif) ligand 1
DAPI: 4',6-Diamidino-2-phenylindole
DIM: Days in milk
DMI: Dry matter intake
ELISA: Enzyme-linked immunosorbent assay
FASN: Fatty acid synthase
FFAs: Free fatty acids
IFN-γ: Interferon-γ
IκB: Inhibitor of nuclear factor kappa B kinase subunit beta
IL-1β: Interleukin-1β
IL-10: Interleukin-10
KLF4: Krueppel-like factor 4
LBP: LPS-binding protein
LIPE: Lipase E
NEB: Negative energy balance
NEFA: Non-esterified fatty acids
NFκB: Nuclear factor kappa B subunit
NLRP3: NLR family pyrin domain containing 3
NOS2: Inducible nitric oxide sythase
OCT: Optimum cutting temperature
p-LIPE: Phosphorylated lipase E
p-IκB: Phosphorylated inhibitor of nuclear factor kappa B kinase subunit beta
PPARγ: Peroxisome proliferator activated receptor gamma
p-NFκB: Phosphorylated nuclear factor kappa B subunit
p-SAMD3: Phosphorylated sterile alpha motif domain containing 3
SAMD3: Sterile alpha motif domain containing 3
SCK: Subclinical ketosis
SEM: Standard error of the means
TAG: Triacylglycerol
TBST: Tris buffered saline tween
TGFβ: Transforming growth factor beta
TLR4: Toll-like receptor 4
TNF-α: Tumor necrosis factor-α
TREM2: Triggering receptor expressed on myeloid cells 2
