# Supplementary material for: Proinflammatory polarization of adipose tissue macrophages in cows with subclinical ketosis constitutes a critical driver of adipose tissue remodeling and inflammation
Source: J Anim Sci Biotechnol. 2025 Sep 29;16:130. doi: 10.1186/s40104-025-01252-3 (PMC12477806; doi:10.1186/s40104-025-01252-3)
Supplement: Supplementary file 1 — Additional file 1: Table S1 Ingredient and nutrient composition of the diets. Table S2 Primer sequences of the genes. [file 40104_2025_1252_MOESM1_ESM.docx]

**Table S1** Ingredient and nutrient composition of the diets

| **Feed composition** | **Value** |
| --- | --- |
| Ingredient, % of DM |  |
| Corn silage | 23.60 |
| Corn | 21.11 |
| DDGS | 4.21 |
| Soybean meal | 9.68 |
| Soybean husk | 3.96 |
| Soybean | 2.10 |
| Cotton meal | 1.89 |
| Alfalfa | 18.8  Oat hay |
| Oat hay | 2.48 |
| Sugar beet pulp | 3.96 |
| Cottonseed | 5.06 |
| Premix^1^ | 2.31 |
| NaHCO_3_ | 0.63 |
| MgO | 0.21 |
| Nutrient content, % of DM  NEL, (mCal/kg) |  |
| NE_L_, Mcal/kg | 1.70 |
| CP | 17.8 |
| NDF | 30.5 |
| ADF | 18.0 |
| NFC | 39.5 |
| Starch | 23.5 |
| Ca | 0.74 |
| P | 0.42 |
| EE | 4.10 |

^1^One kilogram of premix contained the following: Co, 4 mg; Cu, 245 mg; Mn, 982 mg; Zn, 1340 mg; I, 16mg; Se, 7 mg; Fe, 356 mg; Mg, 6mg; Vitamin A, 142,000 IU; Vitamin D, 77,600 IU; Vitamin E 1900 IU

**Table S2** Primer sequences of the genes

| **Gene** | **Primer (5’ to 3’)** | **Accession number** | **Length, bp** |
| --- | --- | --- | --- |
| *CD9* | Forward: CCCCGTTTCACATCAGTCCA  Reverse: ATGCTCTTGGTCTGCGAGTC | XM_027541088.1 | 175 |
| *CD68* | Forward: CTACCACCACCAGTCATCAGAATAC  Reverse: TGTAGTCTCCTATTGCCTCCTTGG | NM_001045902.1 | 150 |
| *CD45* | Forward: AGTGTACCTGGACACCACCT  Reverse: GGGCTTGTAACACCACCGTA | XM_010813547.4 | 162 |
| *TREM2* | Forward: GCTGCGGAATCTTCAAACCC  Reverse: CTGGTAATCACGGGGGTCAG | NM_001079580.2 | 121 |
| *CD86* | Forward: TCAAAGACACAGGCTCGTATCAATG  Reverse: CAGCACTATCAGGTCAGAACTCATC | XM_005201387.5 | 95 |
| *CCL2* | Forward: CTGCAACATGAAGGTCTCCG  Reverse: TGTATAGCAGCAGGCGACTT | XM_027518983.1 | 118 |
| *NOS2* | Forward: CTGGAGGTGCTGGAGGAGTTC  Reverse: CCTGGAGGAGCTGATGGAGTAG | XM_059876394.1 | 105 |
| *IL1B* | Forward: GGCTGATGACCCTAAACAGATGAAG  Reverse: ACGATGACCGACACCACCTG | NM_174093.1 | 135 |
| *CD206* | Forward: ATTGAAGTTTGAAGGCAGTGAAAGC  Reverse: AAGAGTTCGGCATCCTGTTGTTG | XM_003586772.6 | 135 |
| *KLF4* | Forward: CCTGCTCACGACTTTCCCTT  Reverse: GGCATGAGCTCTTGGTAATGG | NM_001105385.1 | 209 |
| *IL10* | Forward: CCTGGAAGAGGTGATGCCAC  Reverse: TGTTTTCGCAGGGCAGAAAG | NM_174088.1 | 134 |
| *ARG1* | Forward: ATGTGGTGGCAGAAGTCAAGAAG  Reverse: GAGTGTTGATGTCCGTGTGAGC | NM_001046154.1 | 150 |
| *GAPDH* | Forward: CCTGCCCGTTCGACAGATAG  Reverse: ATGGCGACGATGTCCACTTT | NM_001034034.2 | 153 |
| *β-actin* | Forward: GCCCTGAGGCTCTCTTCCA  Reverse: GCGGATGTCGACGTCACA | NM_173979.3 | 101 |
